# The global burden of atrial fibrillation: Voices from Asia and Brazil

**DOI:** 10.1016/j.hroo.2024.08.007

**Published:** 2024-08-21

**Authors:** Gregory Y.H. Lip, Uma N. Srivatsa, Jeanne E. Poole

**Affiliations:** 1 Liverpool Centre for Cardiovascular Science at University of Liverpool, Liverpool John Moores University and Liverpool Heart & Chest Hospital, Liverpool, United Kingdom; 2 Danish Center for Health Services Research, Department of Clinical Medicine, Aalborg University, Aalborg, Denmark; 3 Division of Cardiovascular Medicine, University of California, Davis, Davis, California; 4 Division of Cardiology, University of Washington, Seattle, Washington

Atrial fibrillation (AF) is the commonest heart rhythm disorder, and confers substantial mortality and morbidity from stroke, heart failure, dementia, and hospitalizations. In the past, much of the clinical epidemiology of AF has been derived from Western populations, but over the last decade, increasing numbers of studies are evident from non-Western cohorts, particularly from Asia. Even from the main studies emerging from these non-Western countries, there has been much focus on urban data or specialist centers, where AF screening and data from claims or administrative datasets have informed some of the contemporary insights into AF care.

Having a global perspective of AF is highly relevant given ethnic differences in stroke and bleeding rates, as has been highlighted in recent comparative epidemiology studies.^1^^,^^2^ Indeed, the Heart Rhythm Society Global Relations Committee encouraged insights into the global burden of cardiac arrhythmias, with the appointment of committee members from different regions of the world.

One recent initiative was to commission a short series, Global Voices on AF, with 3 insightful articles from Brazil, China, and South Asia (Thailand, India, Sri Lanka), highlighting the challenges associated with AF awareness, detection, and management in these regions, especially in rural communities.^3^, ^4^, ^5^ Unsurprisingly, managing AF in rural communities, especially from low-income countries, presents several challenges. Our series highlights some common themes.

The first article by Santos and colleagues^5^ illustrates how rural communities in Brazil often have fewer healthcare facilities and resources compared with urban areas. For example, in Amazonia region of Brazil, rural communities are often in sparsely populated territories characterized by dense forest. Communities are often located alongside rivers (*ribeirinha*), and geographical barriers mean that travel to the nearest healthcare unit can take many hours to days depending on the season. The problem is compounded by language barriers, with over 300 languages spoken across 400 indigenous groups. From the AF perspective, important challenges in delivery of cardiovascular health due to delayed diagnosis, limited treatment options, and access to specialized care for AF management are highlighted. While telemedicine can help bridge the gap, limited Internet connectivity and technological infrastructure can hinder its effectiveness in managing AF remotely.

Poverty and lower health literacy in rural communities of low-income countries, which can also impact patients' ability to afford medications, adhere to treatment plans, and understand the importance of managing AF effectively. Rural areas may lack community resources such as support groups, rehabilitation programs, and educational initiatives focused on AF management. This can hinder patients' ability to engage in self-care and make informed decisions about their health.

Hence, the second article by Krittayaphong and colleagues^4^ highlights some of these issues and summarizes the scenario in Thailand, India, and Sri Lanka. In India and Sri Lanka, the article illustrates the challenges of AF awareness and detection in these low/middle-income countries. Through initiatives like the community engagement and involvement program in Sri Lanka,^6^ proactive patient and stakeholder engagement had led to development and mapping of patient pathways and streamlined screening, assessments, and integrated care management based on the Atrial fibrillation Better Care (ABC) pathway, at least in the Northern Province of Sri Lanka.^7^ Efforts such as the National Institute for Health and Care Research–funded Global Health Research Group on AF have helped build research capacity, leading to further work in the AF sphere.^8^

While education and awareness about AF among both healthcare providers and the general population in low-income countries may be an issue, this can be addressed by greater patient pathways and education programs. For example, the IMProve treatment with AntiCoagulanTs in patients with atrial fibrillation (IMPACT-AF) trial demonstrated how educational intervention in low/middle-income countries improved knowledge and uptake of oral anticoagulation (OAC), which led to lower strokes.^9^

With regard to the scenario in Thailand, various insights from the comprehensive COhort of antithrombotic use and Optimal INR Level in patients with non-valvular Atrial Fibrillation in Thailand (COOL-AF) registry program address patient risks and management issues.^10^, ^11^, ^12^ Take stroke prevention, for example: in many Western countries, direct oral anticoagulants (DOACs) are the preferred option for stroke prevention in AF.^13^ Globally, vitamin K antagonists (VKAs) (eg, warfarin) are widely used, and are often the first option for OAC, as DOACs are not reimbursed. This focus on VKAs raises the challenge of adequate anticoagulation control, as reflected by the time in therapeutic range.^14^ In some Asian countries, there is also the perception that they have to use a lower international normalized ratio range (1.6–2.5), although the evidence does not support this.^15^ Even with DOAC use, many Asian countries tend to use off-label underdosing,^16^ as Asian patients are perceived to be more likely to bleed compared with non-Asians, with any OAC, whether VKA or DOAC.

Initially, when DOACs were first introduced, the affordability of treatments was also a major issue, especially because DOACs were “self-pay” in many countries. However, the more recent introduction of generic DOACs has partially mitigated the issue. Nevertheless, the cost of medications, procedures, and follow-up care for AF can still be prohibitive for many AF patients in low-income countries, contributing to poor treatment adherence and outcomes.

In China, innovative studies including smart wearables for AF screening (the Huawei Heart Study)^17^ and a cluster randomized trial delivering ABC pathway–based care using mHealth (the MAFA-II mobile Atrial Fibrillation App-II trial [mAFA-II trial])^18^ have been conducted, and these have informed recent guidelines for management of AF in the elderly.^19^ However, the third Global Voices article by Li and colleagues^3^ highlights the scenario in rural villages in China. AF care in large urban centers such as Beijing, Shanghai, and Guangzhou is highly sophisticated and advanced. In contrast, rural China depends on “village doctors” (previously called “barefoot doctors”), many of whom do not have a medical qualification. The work from the Jiangsu Province Rural Community AF Project^20^ investigated screening for AF and the MIRACLE-AF (a novel model of integrated care of older patients with atrialfibrillation in rural China) trial used these village doctors to perform a cluster randomized trial comparing delivery of integrated care management based on the ABC pathway supported by telemedicine with usual care.^21^ Results were presented as a late breaking clinical trial at the 2024 European Society of Cardiology congress in London, showing a clear benefit of ABC pathway on adverse clinical outcomes.

Addressing these challenges in managing AF in low/middle-income countries, especially where there are many rural communities, requires a comprehensive multifaceted approach that includes raising awareness about AF, improving healthcare infrastructure, increasing access to telemedicine services, providing education and support to patients and healthcare providers, and implementing strategies to overcome barriers related to distance, transportation, and socioeconomic factors.

*Heart Rhythm O*^*2*^ is proud to give a global voice to these issues.

## Disclosures

The authors have no conflicts to disclose.
